# Pathogens and planetary change

**DOI:** 10.1038/s44358-024-00005-w

**Published:** 2025-01-15

**Authors:** Colin J. Carlson, Cole B. Brookson, Daniel J. Becker, Caroline A. Cummings, Rory Gibb, Fletcher W. Halliday, Alexis M. Heckley, Zheng Y. X. Huang, Torre Lavelle, Hailey Robertson, Amanda Vicente-Santos, Ciara M. Weets, Timothée Poisot

**Affiliations:** 1 Department of Epidemiology of Microbial Diseases, Yale University School of Public Health, New Haven, CT, USA; 2 Département de Sciences Biologiques, Université de Montréal, Montréal, Quebec, Canada; 3 School of Biological Sciences, University of Oklahoma, Norman, OK, USA; 4 Centre for Biodiversity & Environment Research, Department of Genetics, Evolution and Environment, University College London, London, UK; 5 Department of Botany and Plant Pathology, Oregon State University, Corvallis, OR, USA; 6 Department of Biology, McGill University, Montréal, Quebec, Canada; 7 School of Life Sciences, Nanjing Forestry University, Nanjing, China; 8 Center for Global Health Science and Security, Georgetown University, Washington, DC, USA

## Abstract

Emerging infectious diseases, biodiversity loss, and anthropogenic environmental change are interconnected crises with massive social and ecological costs. In this Review, we discuss how pathogens and parasites are responding to global change, and the implications for pandemic prevention and biodiversity conservation. Ecological and evolutionary principles help to explain why both pandemics and wildlife die-offs are becoming more common; why land-use change and biodiversity loss are often followed by an increase in zoonotic and vector-borne diseases; and why some species, such as bats, host so many emerging pathogens. To prevent the next pandemic, scientists should focus on monitoring and limiting the spread of a handful of high-risk viruses, especially at key interfaces such as farms and live-animal markets. But to address the much broader set of infectious disease risks associated with the Anthropocene, decision-makers will need to develop comprehensive strategies that include pathogen surveillance across species and ecosystems; conservation-based interventions to reduce human–animal contact and protect wildlife health; health system strengthening; and global improvements in epidemic preparedness and response. Scientists can contribute to these efforts by filling global gaps in disease data, and by expanding the evidence base for disease–driver relationships and ecological interventions.

## Introduction

One feature of the Anthropocene is a planetary dysbiosis, in which ecological relationships between hosts and microorganisms shift suddenly, with generally adverse consequences for human, animal and ecosystem health. Infectious disease outbreaks and cross-species transmission events are naturally occurring phenomena. But, as with changes in climate and biodiversity over the past several hundred years, the growing diversity and burden of emerging infectious diseases fall outside historical baselines^1,2^. For most of human history, pandemics were ‘once in a century’ events; since the start of the twentieth century, ten distinct pandemics have occurred, including two in the past fifteen years (Box 1). Every year, several new viruses reach human populations^3^, and the frequency of high-impact pathogen spillover events increases by an estimated 5% and resulting mortality increases by 9%^4^. Non-human animal populations are also increasingly vulnerable to emerging diseases, with epizootics and panzootics of diseases such as chytridiomycosis leading to unprecedented waves of extinctions.

**Fig. 1:**
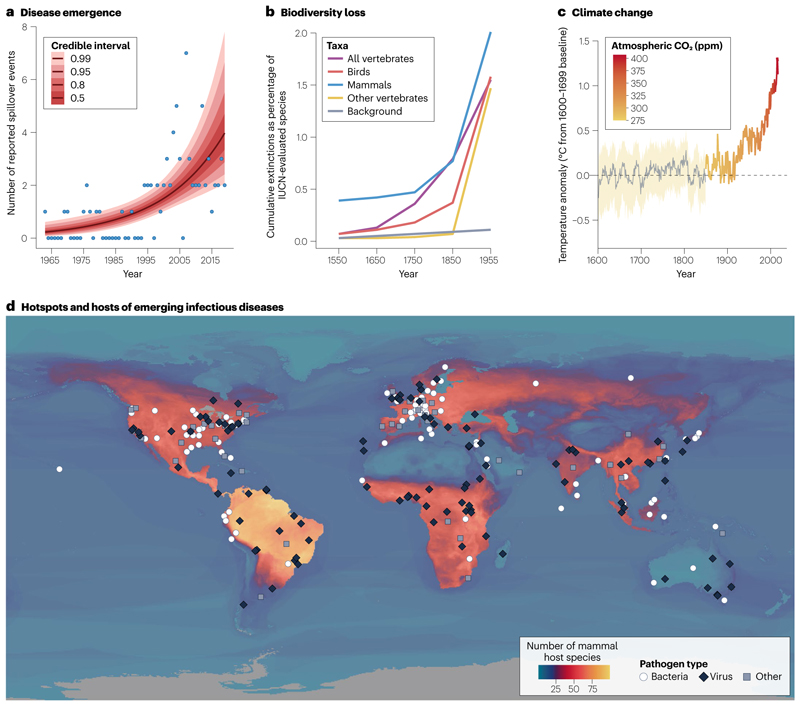
Temporal trends and hotspots. **a**, The annual number of spillover events of high-consequence zoonotic diseases increased steeply during the twentieth century^4^, diverging from historical baselines, similar to the temporal trends in species extinctions^241^ (**b**) and climate change (**c**). However, the apparent trend in spillover rates could be at least partly attributed to improvements in outbreak detection and reporting. **d**, Reports of emerging infectious diseases in humans (data points are coded by pathogen type) are more common in regions of the world with higher mammal biodiversity (number of zoonotic host species); however, outbreaks are also more likely to be detected by surveillance systems and described for the first time in Europe and North America compared to other regions. The effects of anthropogenic environmental change are felt worldwide, and — although high-biodiversity regions face unique risks — the threat posed by emerging infectious diseases is growing everywhere. For a full explanation of data sources and each specific component of the figure, see Supplementary Note 2.

The rising burden of emerging infectious diseases is one of many concurrent and interconnected human-induced changes in the biosphere^1,5,6^ (Fig. 1). Infectious disease emergence, biodiversity loss and anthropogenic global warming have all shown similar trends over the last few centuries. Global hotspots of emerging infectious diseases appear to follow classical biodiversity gradients: new zoonotic and vector-borne diseases have emerged at the fastest rate where mammal biodiversity is also high^7^. However, within ecological communities, loss of host and symbiont biodiversity can also increase pathogen transmission. Biodiversity loss and disease emergence also share many upstream drivers, including agricultural expansion, habitat loss, wildlife trade and climate change. Together, these changes form a ‘polycrisis’ — an interconnected web of rapidly accelerating transformations with no singular solution. The COVID-19 pandemic put these connections in the global spotlight to an unprecedented degree^8–10^. In its wake, some progress has been made towards multilateral action on biodiversity conservation and pandemic prevention, reflecting wider recognition of the links between biodiversity, sustainability and human health. However, planetary transformations have mostly continued as usual, and the risk of future pandemics continues to grow.

In this Review, we synthesize current knowledge on the connections between biodiversity loss and disease emergence, their shared anthropogenic drivers, and their consequences for future epidemic and pandemic risks. We first introduce ecological perspectives on the multi-faceted relationship between biodiversity and infectious disease, including evidence that biodiversity loss can be a risk factor for zoonotic disease emergence. We next present a public health–oriented perspective on how biodiversity science can be used to monitor and manage infectious diseases, and discuss open challenges associated with pandemic prevention through biodiversity conservation. We conclude by describing how future work can clarify the connections between anthropogenic environmental change and infectious disease dynamics, to determine which nature-based solutions could reduce the risk of pandemics.

## Biodiversity and infectious disease

Despite decades of calls for interdisciplinary frameworks and synthesis, ecological perspectives on infectious disease remain relatively fragmented. Macroecologists and systematists consider parasites and pathogens to be part of the sum total of biodiversity, documenting patterns in where and how parasite biodiversity has accumulated^11,12^. Community ecologists explore how species interactions, biodiversity gradients and even biodiversity loss shape disease dynamics over space and time^13^. Some conservation biologists study emerging infectious diseases as a growing threat to species survival, whereas a smaller community of practice is working to save the other >99% of parasites that are mostly harmless^14,15^. The One Health perspective bridges conservation biology, veterinary medicine and public health, focusing on strategies to reduce infectious disease risks at the interfaces among wildlife, livestock, companion animals and humans. Meanwhile, the planetary health approach emphasizes the connections among the climate crisis, the sixth mass extinction and emerging infectious diseases — and how these trends will continue to feed into each other over the coming century. Each of these perspectives paints a slightly different picture of emerging infectious diseases, the role of anthropogenic drivers and the possible ecological levers for intervention. In this section, we summarize the major perspectives on the multi-faceted relationship between biodiversity and disease.

### Host biodiversity drives pathogen biodiversity

Pathogens and parasites account for a substantial fraction of global biodiversity. Parasitism has evolved over 200 times in at least 15 animal phyla^16^: there are hundreds of species of ticks, thousands of species of fleas, tens of thousands of species of parasitoid wasps^17^, several hundred thousand species of worms (a polyphyletic group) that parasitize vertebrate hosts^18,19^, and several million more worms and mites that parasitize invertebrates^20^. The diversity of fungal, bacterial and viral microparasites is even more vast, but harder to quantify; microorganisms are difficult to classify into discrete species, and many switch between mutualist, commensal or pathogenic states depending on their host’s microbiome composition, immune function or ecological context.

The relationship between host and parasite or pathogen diversity is scale- and system-dependent. At broad geographic and taxonomic scales, host species richness is tightly and positively correlated with parasite species richness^21,22^. This ‘diversity begets diversity’ effect is the result of both ecological and evolutionary processes. Any given parasite has a finite intrinsic host range, so on average, more diverse host communities can contain more possible parasites. Over time, parasites also diversify through a mix of cospeciation and host-switching, both of which are facilitated by host diversification^22–24^. However, within a given ecological community or region, the effect of host richness on parasite richness might be secondary to host evolutionary history, host traits (such as body size or immune phenotypes), and environmental conditions (such as climate or ecosystem type)^25,26^.

On the basis of these principles, tropical hotspots of vertebrate biodiversity are also presumed to be hotspots of parasite and pathogen biodiversity. However, the available data are mostly unfit for testing this hypothesis, given both the small fraction of parasite diversity that has been characterized, and the geographic and taxonomic biases of the underlying research (Box 2). For example, macroparasite discovery has been heavily biased towards high- and middle-income countries that invest in systematics research and collections infrastructure; the observed hotspots therefore primarily reflect research effort and capacity^18,27–29^. Viral discovery has been similarly biased towards high-income countries, but it has also been heavily shaped by public health priorities — especially large-scale investments in characterizing viruses with zoonotic potential^30,31^. As aresult, sampling efforts have been idiosyncratic, with a sizeable gap in the Amazon basin and more broadly Latin America, compared to sub-Saharan Africa and southeast Asia.

### Biodiversity drives disease emergence

The vast majority of animal pathogens will never pose a risk to human health, but a small fraction have the capacity to infect humans, given the opportunity. Over evolutionary timescales, this is the origin of almost all human infectious diseases, with very rare exceptions^32,33^. More than 70% of emerging infectious diseases have spread from animals to humans within the past several hundred years, with more than half coming from wildlife (as opposed to livestock or pets). Among emerging viruses specifically, almost 90% are zoonotic, and roughly two-thirds are the result of spillover from wildlife^33^. (On this point, many sources use incorrect citations, statistics, or database^250^ both (Supplementary Note 1)). Viruses pose a unique and ongoing risk as potential zoonotic pathogens, because of their pace of diversification, propensity to cross species barriers, and potential to cause devastating epidemics starting from a single human case. Over 500 virus species have been recorded infecting both animal and human hosts^34^, but tens or hundreds of thousands of mammal viruses (and a small number of other vertebrate viruses) could be capable of human infection^18,35^.

Some animal groups seem to host a disproportionate number of known or potential zoonotic pathogens. One proposed explanation for these apparent ‘hyper-reservoirs’ is that some animal clades could harbour a higher overall pathogen diversity than others: for example, bats comprise 22% of mammals but host 35% of known mammal viruses, whereas rodents comprise 36% of mammals, but only account for 19% of their viral diversity. However, these patterns are nearly impossible to separate from sampling effort, especially in the case of bats, which have been uniquely targeted in virus surveillance efforts since the emergence of SARS-CoV in 2002 (refs. 31,36). Although per-species viral richness is similar across mammal orders^22,37^, some specific clades have higher-than-expected pathogen diversity; this could be a random outcome of evolutionary history, or the result of specific ecological traits, such as a fast pace of life or larger geographic range^38^.

Some animal clades also host pathogens with unique characteristics that increase their potential impact on human populations. Bats have particular immune adaptations that appear to facilitate an exceptional tolerance of virulent viruses, such as constitutive expression of IFNα and a dampened inflammatory response^39,40^. These traits create strong selective pressures on their pathogens^41^, potentially driving features that include a higher propensity for cross-species transmission and a higher intrinsic virulence — explaining why bats host many of the most virulent zoonotic viruses^42^. Similarly, viruses that are adapted to primate immune systems can be functionally ‘pre-adapted’ to humans, and so are more likely to be transmitted onwards after the first human case^42–44^.

At a global scale, the first records of new infectious diseases show a striking correspondence to mammal biodiversity gradients (Fig. 1d), with more newly described diseases in regions with medium to high mammal diversity. Novel infectious diseases are also more likely to be detected by surveillance systems and characterized by researchers in high-income countries, especially in North America and Europe; after adjusting for these biases, the correlation with biodiversity gradients becomes even stronger^7,33^. The association between higher host biodiversity and higher rates of disease emergence results from a higher underlying diversity of the pathogen community, as well as the large number of people and livestock living alongside biodiverse ecosystems^45^. However, there are exceptions to this pattern: most notably, the Neotropics should be a global hotspot of pathogens with zoonotic potential given their high mammal biodiversity, but novel epidemic viruses seem to emerge from wildlife very rarely in Latin America and the Caribbean^46^. This absence is particularly notable in the case of bats, which are most biodiverse in the Neotropics; based on the biogeography of clades that are tightly associated with high-consequence zoonotic viruses, spillover risk should be high in the Amazon basin^37,47–49^. However, to our knowledge, no epidemic of a bat-origin virus has been recorded in South America. These kinds of idiosyncrasy in the relationship between host biodiversity, pathogen biodiversity and disease emergence could be the result of specific coevolutionary history — for example, filoviruses (including Ebola and Marburg virus), henipaviruses (including Nipah and Hendra virus) and SARS-like coronaviruses have all been detected in bats in the Old World but not in the Neotropics (but see refs. 50,51) — or could be due to different socioecological pressures on, and pathways for, emergence (for example, bats might be less regularly consumed for protein in the Americas, and wildlife farming is less common than in east and southeast Asia).

### Biodiversity loss can drive disease emergence

One of the most extensive debates in contemporary ecology revolved around whether biodiversity has a protective effect against infectious diseases^52^. This debate has been mostly resolved through an overwhelming body of empirical evidence and several meta-analyses. In general, differences between communities in baseline host biodiversity (in other words, natural biodiversity gradients) have an inconsistent — and, at the broadest spatial scales, often positive — effect on disease risk. However, the loss of host biodiversity within a given ecological community is typically followed by an increase in pathogen transmission^53,54^. This finding has been reproduced in observational and experimental studies, with terrestrial and marine systems, animal and plant hosts, directly transmitted and vector-borne pathogens, and different types of infectious agent^54,55^. However, in any given instance, this pattern could be the result of indirect association (biodiversity loss and disease emergence may share a driver such as habitat loss) or direct causation (biodiversity loss may directly increase pathogen prevalence in wildlife or spillover rates).

Biodiversity loss does not favour every pathogen or parasite equally: some decline or are lost alongside their hosts, whereas others become more prevalent as their hosts or vectors become more abundant. The fate of any given pathogen depends on the host competence of each available host species, their interactions with each other and their responses to anthropogenic disturbance. In general, anthropogenic change will increase disease risk if it favours host species that are important to pathogen transmission, or if it leads to the loss of other species. For example, loss of a keystone predator might lead to larger and more connected prey populations, increasing pathogen transmission (the ‘healthy herds effect’)^56–58^. Similarly, if environmental changes lead to the disproportionate loss of low-competence hosts (namely, those that serve as a sink or dead end for transmission), prevalence can increase in the remaining species (the ‘dilution effect’)^59,60^. For example, forest fragmentation favours the white-footed mouse (*Peromyscus leucopus*)^61^, which is a highly competent host for both the bacterium that causes Lyme disease and the ticks that transmit it; meanwhile, lower-competence hosts, such as opossums, become less abundant in disturbed landscapes^62^. The universality of the dilution effect and its relevance to human health have been heavily debated, but across ecosystems, the species that are most resilient to anthropogenic change are also more likely to be hosts of zoonotic diseases^38,63^. There are several explanations for this pattern, but many focus on life history: fast-lived animals that thrive in disturbed environments (‘weedy’ species such as the white-footed mouse) often undergo explosive population cycles that create episodes of high spillover risk^64^, and might also be subject to evolutionary trade-offs in immune investment^65–67^.

Not all parasites respond positively to anthropogenic change. Parasites are vulnerable to the loss of their hosts^68–70^, but can also be directly affected by environmental stressors that influence transmission or survival, especially in their free-living stages^71–73^. Paradoxically, decreases in total parasite richness can be accompanied by increased disease risk from specific pathogens. Sometimes, this decrease occurs because parasites are in direct competition, not just within host populations but within individual hosts (in other words, coinfection leads to worse disease outcomes for the host, limiting parasite transmission at high prevalences)^74,75^. In other cases, complex interactions between parasite infection and host immunity can reduce host susceptibility to infection with a more virulent pathogen^73,76^. Conservation strategies that proactively conserve hosts alongside a diverse parasite fauna could therefore help to protect them from the emergence of diseases that jeopardize their survival or even human health^77^. However, the field of parasite conservation biology, and broader scientific understanding of the ecological consequences of parasite biodiversity loss, is still in its infancy.

### Disease can drive biodiversity loss

Emerging infectious diseases pose a growing problem for wildlife conservation^78^, with high-profile examples of mass mortality resulting from the introduction of novel pathogens or unusual outbreaks of endemic pathogens triggered by changing environmental conditions^79^. For example, during unusually warm and humid weather in 2015, an outbreak of an endemic and usually benign bacterium (*Pasteurella multocida*) in Kazakhstan was responsible for the loss of 60% of the global population of the saiga antelope (*Saiga tatarica*)^80,81^. Only two years later, the virulent peste des petits ruminants virus spread from livestock to saiga in Mongolia, leading to the loss of 80% of the local population^82^.

Epizootics of virulent pathogens are often self-limiting — as the number of susceptible hosts declines, infected hosts eventually die faster than they produce secondary infections — and, in isolation, are unlikely to cause the extinction of an entire species^83^. However, infectious disease can readily reduce wildlife populations to low levels, where they face an increased risk of extinction due to other factors; conversely, small and isolated populations such as island endemic species can be vulnerable to outright disease-induced extinction.

A subset of pathogens can also continue to spread through environmental reservoirs even as host populations reach critically low levels^84^. Indeed, several prominent disease-induced extinctions of wild animals are attributed to fungal pathogens that can persist in the environment. Since the late twentieth century, a panzootic of the chytrid fungus *Batrachochytrium dendrobatidis* has been responsible for the extinction of at least 90 amphibian species^85^. Another chytrid fungus, *B. salamandrivorans*, has been responsible for mass mortality events in some European salamander populations^86^ and could someday become a similarly global problem^87^. The fungus responsible for white-nose syndrome (*Pseudogymnoascus destructans*) has similarly led to the collapse of North American bat populations, with at least one species still at risk of extinction^88^. These pathogens have had unexpected repercussions for human health: the loss of Neotropical amphibians that feed on mosquito larvae might have increased malaria incidence in Costa Rica and Panama^89^, and the loss of insectivorous bats might have forced farmers to use more insecticide, leading to higher rates of infant mortality in the eastern USA^90^.

Beginning in 2020, highly pathogenic avian influenza A/H5NX sublineage 2.3.4.4b has been responsible for numerous mass mortality events in wild birds and mammals^91^, representing a potentially unprecedented panzootic threat. In 2023, 27% of the total population of Chilean Humboldt penguins (*Spheniscus humboldti*) were found dead, representing a nearly 2,000% year-to-date increase in mortality^92^. After several critically endangered California condors (*Gymnogyps californianus*) died from avian influenza in 2023, rapid efforts to develop an emergency vaccine were initiated.

Human pathogens also pose a growing risk to wildlife health. Human-to-animal pathogen spillback is limited by the same ecological and evolutionary bottlenecks as zoonotic spillover, as well as the asymmetry of many human–animal interactions (for example, humans eat other animals at a much higher frequency than the inverse). However, just as spillover rates are growing exponentially, spillback could also be a growing problem. This phenomenon is perhaps most visible in the global spread of SARS-CoV-2, which has been found in 35 animal species across 5 continents^93^. Whereas SARS-CoV-2 has generally had minimal effects on conservation, in other cases, the outcomes of pathogen spillback have been serious. For example, primates appear to be more vulnerable to multiple human infections than other mammals due to their evolutionary proximity to humans^94^, and respiratory pathogens that are relatively benign in humans (for example ‘common cold’ viruses) regularly cause serious mortality in great apes^95,96^. Primates living in sanctuaries or that become habituated to human landscapes seem to be particularly at risk^94^, but the effect on natural populations can still be substantial.

Conservation measures that limit human–wildlife contact are the primary defence against pathogen spillback. Surveillance for wildlife mortality and active pathogen surveillance are especially important where humans live alongside wildlife species or are expanding their reach into critical habitat for threatened species. Investment in human and livestock health around protected areas can also limit the level of exposure that wildlife face^97^. These measures, in turn, reduce the risks and effects of zoonotic spillover in high-biodiversity areas.

## Common drivers and causal pathways

The same anthropogenic processes that are responsible for the biodiversity crisis are also implicated as the primary ecological drivers of disease emergence^9,98–101^. Land and climate change both mediate disease dynamics through organismal physiology and behaviour, and agriculture and wildlife trade and hunting act as distinct high-risk interfaces for animal-to-human pathogen transmission. However, the effect and importance of any given driver of biodiversity loss or disease emergence — and the relationship between the two — can be unique to a given pathogen, and even a given landscape. Putting these connections into context can help researchers to produce better risk assessments and identify points for intervention.

### Upstream drivers

**Fig. 2:**
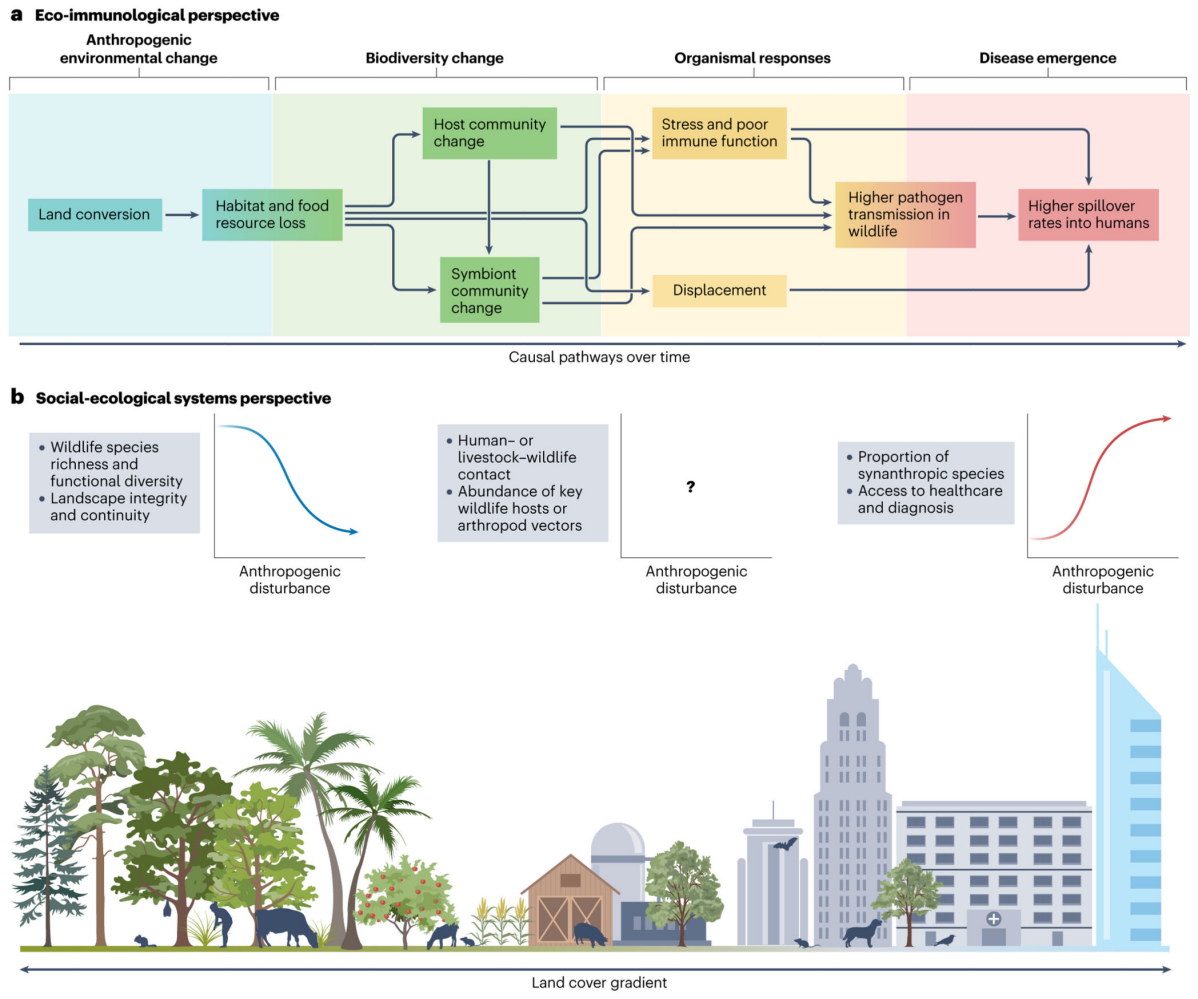
Two perspectives on land use as a driver of biodiversity loss and disease emergence. Biodiversity loss and disease emergence often follow land conversion, but different schools of thought offer different insights into the underlying mechanisms. **a**, In a conceptual model based on ecoimmunology and community ecology^104,105^, land conversion sets off a cascade of organismal and community-level changes; over time, this increases the risk of zoonotic spillover. **b**, In a conceptual model based on landscape ecology and social–ecological systems theory^63,107^, spillover risk is shaped by the intensity and type of anthropogenic land use, the habitat requirements of important species in the pathogen life cycle, and the types of interface associated with spillover (for example, wildlife hunting or agriculture). Some variables are usually positively or negatively correlated over space with the degree of anthropogenic disturbance; other gradients might be unique to the ecology of a given landscape and pathogen. Neither model is comprehensive or universal, and both are compatible with other perspectives.

Since the 1960s, one-third of global land area has undergone anthropogenic land change^102^, in the form of conversion and fragmentation of intact forests and other ecosystems, of agricultural expansion and intensification, and of urbanization. Habitat loss from land change (and increasingly, climate change) is the single greatest threat to biodiversity^103^. Land change is also often cited as the primary driver of zoonotic spillover^104^. Habitat loss, fragmentation and degradation can cause nutritional stress and behavioural shifts that increase contact within and between species, all of which can lead to increased pathogen transmission^104,105^ (Fig. 2a). Habitat loss also pushes wildlife into human-used landscapes to seek resources, shelter or space^63,106^, leading to increased spillover risk.

The response of a given pathogen to land change depends on its transmission ecology. Human cases of vector-borne zoonoses (such as yellow fever virus) appear to increase more consistently following land-use change than does spillover of directly transmitted pathogens (such as Ebola virus)^5,107^. However, risk can also decrease after land conversion if key wildlife hosts or vectors are poorly suited to human-altered landscapes or are excluded by synanthropic species^54,108,109^ (Fig. 2b). Land conversion is therefore sometimes associated with a regime shift between different assemblages of human pathogens. For example, in Brazil, the transition from rural to urban landscapes is accompanied by a shift from malaria and leishmaniasis to arboviruses such as dengue fever and Zika virus that are primarily transmitted by the *Aedes aegypti* mosquito^109^.

In addition to being the largest driver of deforestation, animal agriculture poses unique risks relative to other types of land use. Of the four facets of planetary change discussed here, agriculture had the earliest (and longest) effect on disease emergence; human and domesticated animals have had thousands of years to share viruses^110^.

Livestock also account for more terrestrial biomass (around 630 million tonnes) than humans (around 390 million tonnes) or wild mammals (around 20 million tonnes)^111^; this abundance creates ample opportunities for pathogen circulation, evolution (including adaptation to mammalian immune systems) and cross-species contact. Livestock therefore often act as bridge or amplification hosts in the disease emergence process^112–114^. For example, in nearly half of all modern pandemics, emerging influenza virus subtypes infected poultry or other livestock before spreading to humans. Livestock can also be a source of pathogens that threaten wildlife populations, such as highly pathogenic avian influenza or tuberculosis^115^.

Compared to land change, climate change is often underestimated as a threat to both biodiversity and human health, both because climate-related risks are still accelerating, and because they can be hard to distinguish from other correlated trends, including improved surveillance. Rising temperatures, shifts in precipitation and severe storms could be responsible for increasing the risk of over half of human diseases^116^. So far, the best understood effect of climate change on disease risk has been an increase in the global burden of mosquito-borne diseases, which exhibit a well characterized unimodal transmission–temperature relationship^117–119^. Climate change is also implicated as a contributing factor in the rapid range expansion of the mosquito vectors of malaria and dengue fever^120,121^ and similar effects on ticks are also suspected, but less clearly established^122,123^. Climate-change-driven geographic range shifts are also creating new opportunities for interspecific contact among wildlife^124,125^, increasing risks related to epizootics and creating potential bridge hosts for zoonotic emergence^126^. In their native ranges, many species are also increasingly exposed to extreme temperatures they have never encountered^127,128^, posing a serious risk to species survival and ecosystem stability; the implications for disease transmission have barely been explored.

A final planetary driver of both biodiversity loss and emerging disease is wildlife use, including trade, farming and hunting. So far, human cases of fewer than 10% of emerging viruses have been traced back to wildlife use^112^, but some of these viruses pose a particularly serious risk — most notably, SARS-like coronaviruses in southeast Asia^129–132^. Wildlife trade affects a quarter of vertebrate species and has become a major threat to the survival of many species^133^. Wildlife farms, supply chains and live-animal markets all create unnatural conditions that can increase crowding and physiological stress, leading to higher rates of infection^134,135^ as well as unusual contact patterns between species^136^. Every stage of the commercial wildlife trade process also entails high-risk contact between humans and animals, and spillover events could be more likely to lead to epidemics if traded wildlife are brought into large population centres. Although generally lower-risk than commercial wildlife trade, subsistence hunting can also threaten species survival^137,138^ and create opportunities for pathogen spillover.

Although land change, agriculture, climate change and wildlife use are the most important ecological drivers of biodiversity loss and disease emergence^54^, several other facets of anthropogenic change are known — in more limited cases — to affect both processes. Invasive alien species are involved in 60% of modern extinctions^139^, and can bring pathogens into new regions. For example, two globally invasive mosquitoes (*A. aegypti* and *Aedes albopictus*) have become the primary vectors of several arboviruses, including dengue fever, yellow fever, chikungunya and Zika virus^121,140^. A third synanthropic mosquito from south Asia (*Anopheles stephensi*) now poses a similar risk of global invasion, and represents a growing threat to malaria eradication in Africa^141,142^. Pesticide pollution can cause ecosystem changes that increase disease risk; for example, fertilizer runoff favours invasive aquatic weeds, which create habitat for the snail vectors of schistosomiasis^143^. Finally, leakage of antibiotics into the environment is a major contributor to the global crisis of antimicrobial resistant bacteria and fungi^144,145^, selecting for the emergence of drug-resistant pandemic viruses before they ever spread from animals to humans^146,147^.

### Case studies in causation

**Fig. 3:**
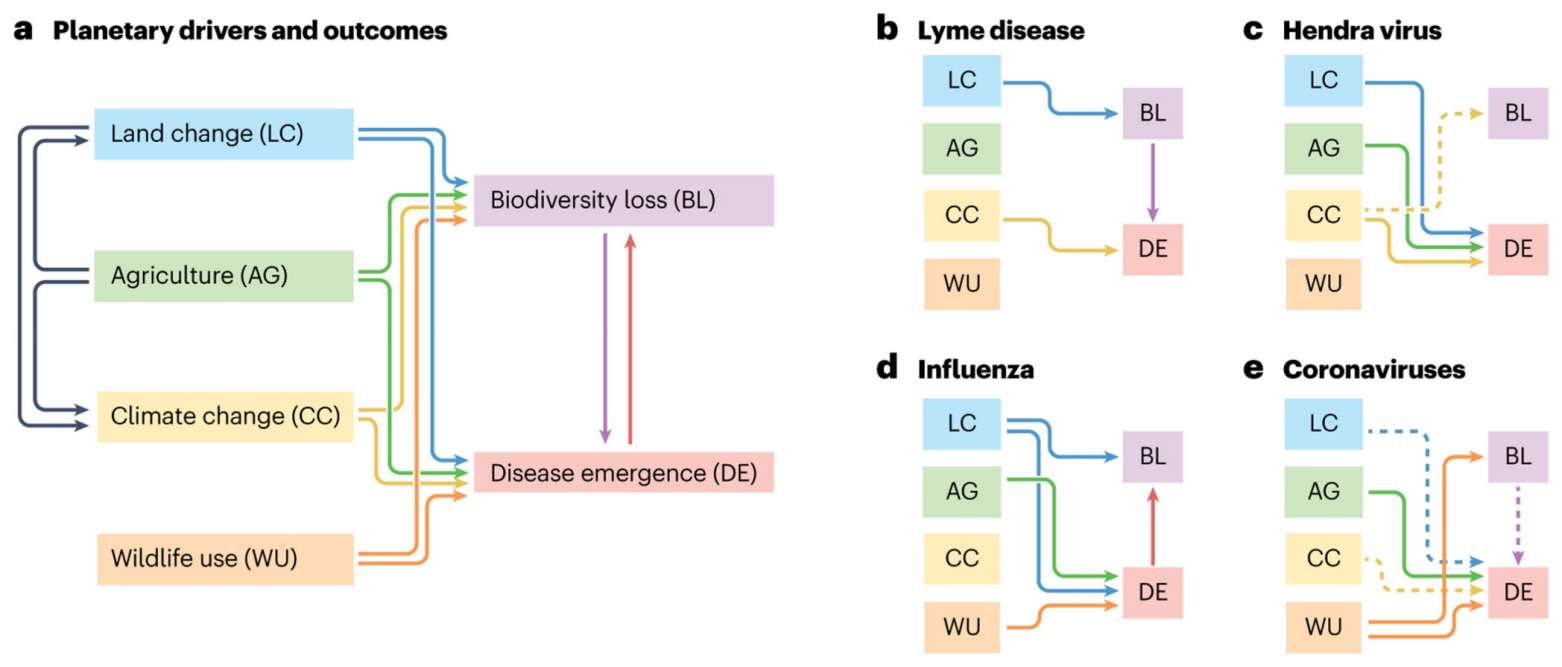
Case studies in biodiversity–disease–driver relationships. **a**, Biodiversity loss can drive disease emergence, and vice versa; they also share many of the same upstream drivers. Three of these facets of anthropogenic global change are closely linked: agriculture is the single largest driver of land change, and agriculture and land change are both major drivers of climate change. The strength and direction of these relationships vary substantially on a disease-by-disease basis; case studies are shown for Lyme disease (**b**), Hendra virus (**c**), influenza (**d**), and coronaviruses (**e**). In **b**–**e**, solid lines indicate relationships supported by direct literature evidence, and dashed lines indicate relationships that are hypothesized but weakly supported, or that are more likely to be important in the future. To mitigate infectious disease risks, scientists need to establish these kinds of relationships, and identify case-by-case interventions that target the right drivers.

There is no canonical biodiversity–disease–driver relationship (Fig. 3). To illustrate the diversity of these interactions, we discuss four case studies that exemplify how variation in pathogen transmission mode, in wildlife host or vector identity, and in anthropogenic context can influence the dynamics of disease emergence.

#### Lyme disease

Lyme disease is caused by the bacterium *Borrelia burgdorferi*, and primarily vectored by the blacklegged tick (*Ixodes scapularis)*. Since it was first identified in 1975, the incidence of Lyme disease has grown substantially, primarily in the northeast and mid-west regions of the USA. Land change and biodiversity loss have contributed to this trend: forest fragmentation has led directly to the loss of low-competence hosts such as opossums while favouring competent reservoirs such as mice, chipmunks and shrews^61,62,148^. Additionally, declines in key predator species such as red foxes have also increased the abundance of these competent hosts^58^. Although climate change has not been the primary driver, the growing burden of Lyme disease in the northeastern USA is at least partially attributable to rising temperatures, and is likely to continue to increase under future warming^149^.

#### Hendra virus

Hendra virus is a pathogen of Australian flying foxes (*Pteropus* spp.); spillover into humans occurs infrequently and does not lead to onward transmission. Human–bat interactions have increased as the bats’ winter habitats have been converted into agricultural land, and previously nomadic populations have settled in urban environments, creating more opportunities for Hendra virus spillover^106^. The loss of nomadic behaviour also reduces population connectivity and therefore allows immunity to wane, leading to larger epidemic sizes upon viral re-introduction^106,150^. Transmission dynamics are also probably affected by climate change. El Niño climate oscillations create years in which fruit resources are insufficient, driving bats into agricultural land to forage; this nutritional stress also increases seasonal pulses of viral shedding^106,151^. Habitat loss from extensive bushfires could lead to similar risks^152^. Finally, extreme heat associated with climate change killed over 72,000 bats, and caused widespread abandonment of pups in the summer of 2019–2020 (ref. 153); the implications of this kind of mortality event for disease dynamics are uncertain.

#### Influenza

Influenza A virus is the archetypal pathogen with pandemic potential and, increasingly, a global threat to biodiversity^91,154^. Viral strains undergo genetic drift and reassortment in poultry and other livestock, which also transmit the virus back to wild birds and humans. Although surveillance and prevention efforts often focus on farms, spillover has also been associated with poultry markets and the wild-bird trade, particularly in China and southeast Asia^155,156^. Anthropogenic drivers of influenza circulation in wild birds are comparatively understudied. The precipitous loss of the world’s wetlands might be forcing migratory waterfowl to congregate in smaller patches of intact habitat, leading to higher levels of transmission^157,158^; protected areas could reduce outbreak risk by reducing this pressure and separating waterfowl from domestic poultry. Despite speculation that climate change could be a contributing factor to the H5NX highly pathogenic avian influenza panzootic^159^, there is so far no evidence to support this hypothesis.

#### Coronaviruses

The *Coronaviridae* are an immensely diverse family of viruses found across mammals and birds, although some groups that have diversified in bats (particularly the subgenera *Sarbecovirus* and *Merbecovirus*) pose a distinct risk to human health. Fewer than a dozen coronaviruses have so far emerged in humans, of which only three have shown both high pathogenicity and pandemic potential: two severe acute respiratory syndrome coronaviruses (SARS-CoV and SARS-CoV-2), and Middle East respiratory syndrome coronavirus (MERS-CoV). The strongest available evidence indicates that SARS-CoV and SARS-CoV-2 both reached humans through the wildlife trade^129,160,161^, which also poses a serious threat to the conservation of suspected wildlife hosts. However, the majority of human coronaviruses — including MERS-CoV, several low-pathogenicity viruses, and the most recent additions, canine coronavirus and porcine deltacoronavirus — are known or suspected to have reached humans through livestock and companion animals^162–164^. Proposed connections between the specific origin of COVID-19 and land change^165^ or climate change^166^ are so far speculative. However, coronavirus prevalence is higher in wildlife-use-related contexts^134,135^ and in ecosystems with a greater human footprint^167^. One study found promising evidence of a dilution effect in the bat–coronavirus system in west Africa^168^, but more work is needed to establish whether interspecific variation in immunology contributes to differences in host competence and whether biodiversity might therefore have a protective effect.

## Shared solutions for biodiversity and health

Within the next 25 years, the world is on track for at least 1.5 °C of warming and nearly 300 million hectares of tropical deforestation^169^. At the same time, based on current trends, a four-fold increase in the rate of zoonotic spillover, with 12 times as many deaths, is projected^4^. These problems call for a diverse set of solutions, ranging in scale from local initiatives^170^ to planetary governance^171,172^. Perspectives from ecology and biodiversity science can be leveraged to develop better surveillance infrastructure and ecosystem-based strategies for outbreak prevention, in tandem with renewed investments in public health and outbreak preparedness and response.

### Biosurveillance and biodiversity monitoring

Surveillance is the backbone of public health. The One Health approach highlights the importance of monitoring pathogens not only in humans, but also in wildlife, domestic animals and environmental reservoirs such as soil, water and air. Given resource limitations, surveillance efforts should target the hosts and interfaces most associated with specific epidemic or pandemic risks, or the major data gaps that limit scientific inference. Machine learning models can also help to target sampling and monitoring efforts towards species that are most likely to host undiscovered pathogens^48,49,173^, are at the highest risk from viral spillback^174,175^, or are likely to display spillover-relevant behaviours such as living in human-built structures^176^.

Several technological advances are improving wildlife disease surveillance. For example, mobile apps such as the Spatial Monitoring and Reporting Tool (SMART)^177^ allow park rangers to report unusual mortality events. Platforms like Verena’s Pathogen Harmonized Observatory^178^ and the United States Geological Survey’s Wildlife Health Information Sharing Partnership^179^ allow researchers and managers to share wildlife disease data in real time. Non-invasive sampling methods also open up new horizons for biosurveillance: for example, air samples collected by drone can be used to monitor marine mammals^180^ or to sample live-animal markets or bat roosts without putting researchers at risk of pathogen exposure^181^. The advent of ‘next-generation biomonitoring’^182–184^, with its explicit adoption of artificial intelligence-enhanced image and sound analysis as well as environmental DNA and RNA data collection, is also increasing the volume and resolution of data that can be collected.

**Fig. 4:**
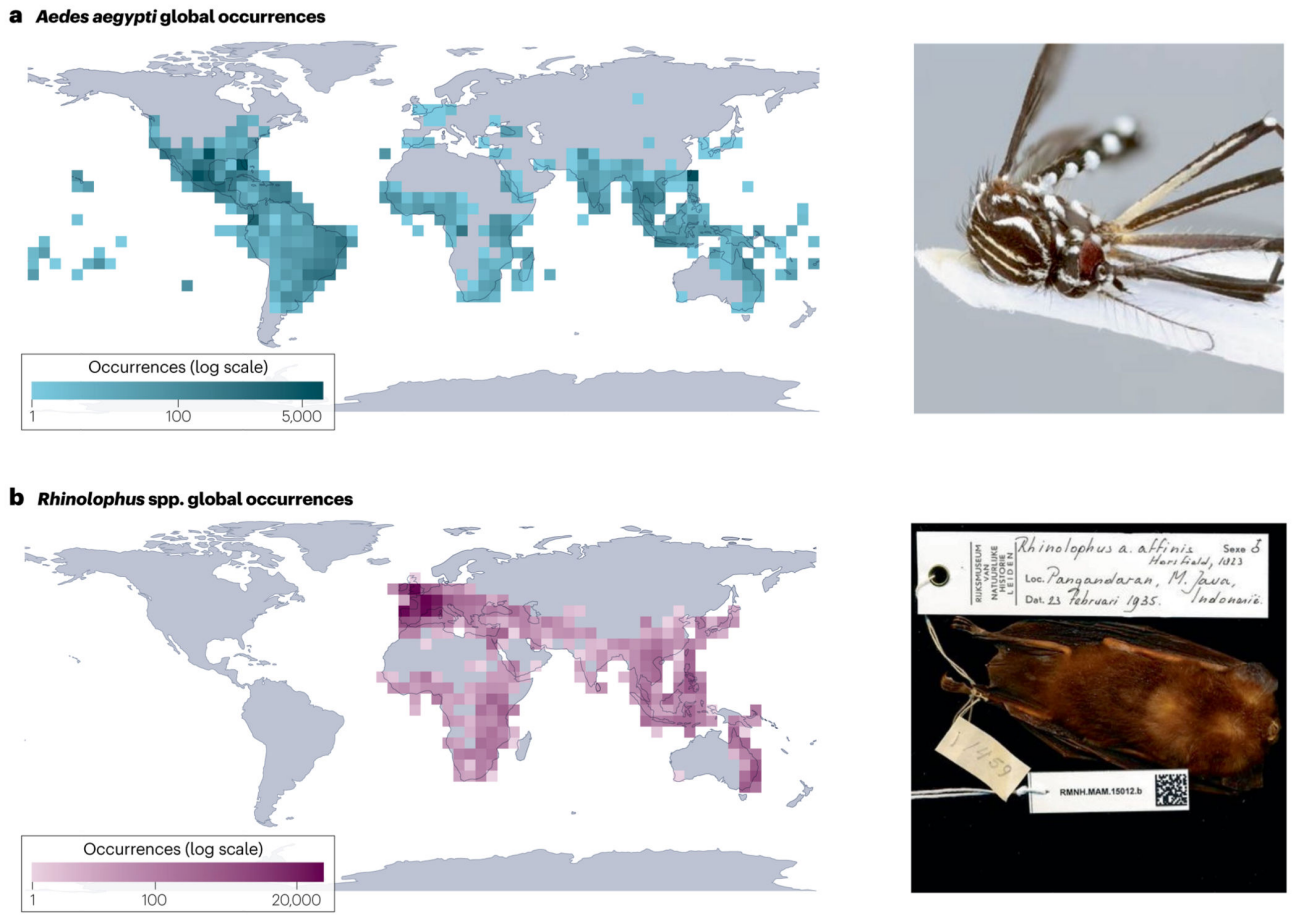
Biodiversity science as biosurveillance. **a**, Geolocated occurrence records for *Aedes aegypti*, the primary urban vector of several viruses, including dengue, yellow fever and Zika. **b**, Geolocated occurrence records for horseshoe bats (*Rhinolophus* spp.), the primary known wildlife reservoir of SARS-like coronaviruses. Information contained in digital biodiversity infrastructure and museum collections is both a foundational resource for long-term ecological research and an open source of real-time epidemic intelligence and viral discovery. The photograph in **a** is reprinted from https://www.gbif.org/occurrence/3910014308, CC0 (https://creativecommons.org/publicdomain/zero/1.0/legalcode); the photograph in **b** is reprinted from https://www.gbif.org/occurrence/2432534405, CC0 (https://creativecommons.org/publicdomain/zero/1.0/). GBIF, Global Biodiversity Information Facility.

Biodiversity science has also become a critical source of data for public-health research and practice^185^. Geospatial data on disease hosts and vectors are regularly used to map disease transmission risk^186,187^, to identify surveillance gaps^188^, to reconstruct historical patterns, such as the spread of invasive vectors^120,121^, and to project future infectious disease risk under different scenarios of changing climate and land use^126,189,190^. Biodiversity repositories are usually the best available source of these data, although microorganisms remain under-represented in major biodiversity data platforms^191^ (Fig. 4a). Targeted efforts to recruit new data^192,193^, particularly from rich sources such as community science projects (for example, the Mosquito Alert app)^194,195^ and vector control agencies^196^, increase the value of these datasets for public health and for biodiversity research more broadly.

Museum collections can also support specimen-based research on infectious diseases^197–199^: by dissecting preserved animals, researchers can track long-term trends in their parasite communities and even test hypotheses about drivers such as climate change^71,200,201^. Preserved tissues can also be used to discover uncharacterized pathogens, including from species that are otherwise hard to sample (for example, rare and endangered species)^202,203^ (Fig. 4b).

### Managing infectious disease risks

Public health and conservation can benefit from interventions that reduce pathogen transmission within wildlife populations or limit opportunities for cross-species transmission at the wildlife–livestock–human interface. Many public-health-oriented spillover prevention strategies include an educational component focused on living safely alongside wildlife^204^, especially when communities might otherwise rely on destructive interventions such as cullings that harm wildlife populations and can inadvertently increase spillover risk^205–207^. Other active strategies include wildlife and livestock vaccination or invasive species control^208^.

Ecologists have also called for ecosystem-based interventions that target the upstream drivers of disease emergence. Examples of these strategies include land-management decisions that preserve intact forests, supported by the preponderance of evidence that forest loss and fragmentation are often followed by an increase in zoonotic spillover risk. Other strategies that aim to reverse ecosystem changes, such as afforestation and reforestation^106,209,210^ or prescribed burns^211^, might achieve similar results, but there is only a small amount of primary research testing this assumption.

Among the range of interventions to restore ecosystems and reduce infectious disease risks, the most successful ‘win–win’ interventions for conservation and human health are those that are motivated by detailed knowledge of system dynamics, often from long-term case studies^106^; that involve locally led design and decision-making, aligned with pre-existing community priorities; and that are low-cost or, even better, aligned with existing economic incentives^208,212,213^. Without these factors, interventions are usually less successful and could have unintended negative consequences for human health, conservation, or both, as in the case of mosquito net fishing^214^, or unsuccessful restrictions on wildlife hunting and live-animal markets^215,216^.

Ecological strategies are only part of an effective strategy to combat emerging infectious diseases. Although popular narratives often frame spillover as the direct consequence of disordered relationships between humans and nature^217,218^, people are also regularly exposed to zoonotic and vector-borne diseases simply by living alongside other mammals, insects and biodiverse ecosystems^107^. The burden and consequences of those infections — namely, disease severity at the individual level and outbreak effects at the population level — are determined as much, if not more, by social, economic and political factors than by any facet of local ecology or global anthropogenic change^219–222^. Alleviating poverty and improving access to healthcare are recognized as prerequisites not only for improving population health, but also for sustainable development and use of natural resources^170,223,224^. Ecological solutions to manage disease risk will therefore be most effective in combination with ‘tried and true’ public health strategies — namely, health system strengthening^225,226^ and capacity building for outbreak preparedness and response^227^.

### Unsolved problems for planetary governance

Despite the connections between biodiversity loss and emerging infectious diseases, global efforts on the two problems have historically run in isolation. Existing multilateral organizations (such as the World Health Organization (WHO)) and agreements (for instance, the International Health Regulations, in 2005) related to human health generally focus on outbreak preparedness and response, with less attention paid to prevention or the environmental determinants of health. Conversely, conservation-related organizations (for instance, the UN Environment Programme) and agreements (for instance, the Convention on Biological Diversity (CBD) in 1992) or the Convention on International Trade in Endangered Species of Wild Fauna and Flora (CITES) in 1973)) often address human health as a priority, but have usually been treated as ancillary to the global health security architecture.

The COVID-19 pandemic put zoonotic diseases and their drivers into the spotlight, with substantial associated changes in global governance. The United Nations’ new quadripartite partnership — a collaboration among WHO, the Food and Agriculture Organization, the World Organization for Animal Health and the UN Environment Programme — has established a One Health High-Level Expert Panel and produced a One Health Joint Plan of Action. This plan calls for improved scientific understanding of disease emergence; integration of human and animal disease surveillance systems, risk-assessment tools and triggers for action; national development of evidence-based legislation; and sustainable financing for One Health programmes. Meanwhile, the CBD secretariat has begun developing a global action plan on biodiversity and health, and the CITES secretariat has entered into a collaborative agreement with the World Organization for Animal Health, aimed at sharing technical expertise on wildlife trade and its risks to human health.

The upstream drivers of disease emergence pose a more complicated problem for global policy action. Biodiversity loss, deforestation, climate change, agricultural intensification and wildlife trade are all continuing to increase, and some experts have suggested that reversing these trends should be the highest priority for pandemic prevention efforts^98,99,101^. International environmental treaties — such as CBD, CITES, and the UN Framework Convention on Climate Change (in 1992), and related treaties (most notably, the Paris Agreement in 2015) — have all made substantial, but incomplete, progress in their respective areas. The growing cost of emerging infectious diseases has strengthened the case for action on environmental issues^98,228^, but these efforts still face an uphill battle against the overwhelming financial interests of extractive industries. Notably, consumer demand and corporate interests in the USA, Europe and China have often been a substantial barrier to the success of these treaties in the rest of the world^229–231^.

In any plausible scenario for global economic development and environmental change, it is unlikely that spillover rates will decrease within the next few decades: without significant improvements in both outbreak prevention and preparedness, epidemics and pandemics will continue to increase in frequency, effects and duration for at least a generation. Efforts to strengthen the global health security architecture are therefore a critical step to preparing for the health effects of anthropogenic environmental change. If adopted, the proposed WHO Pandemic Agreement is likely to acknowledge the One Health concept, and might establish obligations for the parties related to surveillance, workforce and policies aimed at zoonotic disease prevention. However, One Health programmes could cost an estimated US $22.0–31.2 billion per year^98,99^, presenting a barrier to more substantive action. Multilateral strategies to address issues such as wildlife trade are also unlikely to be well developed in the finalized text, but could still be achieved by an annex or protocol to the treaty^232^. Despite formidable challenges, the proposed Pandemic Agreement and the amendments to the revised International Health Regulations adopted in 2024 both represent major steps forward for public health emergency preparedness and response: in tandem, they could create ways to finance capacity building, increase compliance and cross-talk among national governments, and, most importantly, to ensure that vaccines and other countermeasures will be shared more equitably during future emergencies (Box 3).

## Summary and future directions

Linkages between anthropogenic environmental change, biodiversity loss and disease emergence are widespread, and are often strong determinants of human and wildlife health outcomes. However, knowing that these general principles exist is not a substitute for system-specific knowledge. Scientists need to understand the ecological and evolutionary principles that apply on a case-by-case basis, and connect specific evidence to different public health objectives (such as pandemic prevention, reducing the burden of vector-borne disease or managing risks related to bat viruses). Data, evidence and interventions can existin a dynamic feedback loop, supported by collaborations across biodiversity science, disease ecology, epidemiology and public health. In parallel, several important gaps in scientific research and synthesis need to be addressed.

First, disease ecologists need to develop a more taxonomically, geographically and ecologically diverse evidence base around biodiversity–disease-driver relationships. Wildlife disease research is heavily biased toward certain combinations of regions, hosts and pathogens: for example, rabies virus is disproportionately studied, particularly in vampire bats, relative to other bat viruses in the Americas^233^. These biases are both driven by data gaps, and perpetuate them (Box 2). Similarly, habitats with some degree of disturbance are better studied than pristine areas, which makes it harder to measure the effects of disturbance on disease dynamics, compared to balanced sampling designs. Most studies are also limited to single sampling events^234^; longitudinal study designs can document the effects of anthropogenic changes as they unfold, and demonstrate causality with a high degree of confidence, particularly if researchers also collect data that capture organismal responses beyond infection (for example, protein biomarkers of stress and immune function^235^). Most importantly, researchers should make a concerted effort to share raw, reusable, fine-scale spatial data on organism-level infection patterns^236^, particularly in the most data-deficient systems and parts of the world.

Second, more primary research is needed on the effects of biodiversity–disease-driver relationships on human health outcomes. The relationship between ecological change or biodiversity loss and disease outcomes is often found to be weaker in humans than in wildlife^54,237^, presumably because infectious-disease dynamics in humans are mediated by a number of other social and structural factors. An inclusive view of social–ecological systems, and additional comparative evidence across human disease systems, could challenge paradigms based primarily on wildlife studies and well known case studies^107^. Data availability remains the primary challenge: high-resolution data on infectious disease outbreaks are mostly unavailable to researchers, especially at a spatial scale that aligns with ecological processes such as forest clearing or livestock–wildlife contact. Efforts to compile data from the literature, collaboration with health ministries, and improved outbreak surveillance in remote communities would all help to close this gap.

Finally, research priorities in ecology and biodiversity science should be better aligned with public health priorities. Despite being motivated by pandemic prevention, many disease ecology studies focus on pathogen systems that pose a minimal pandemic threat (for example, Nipah virus or Lassa fever), whereas systems with higher risk (for example, influenza or primate viruses) are comparatively understudied. Similarly, more research is needed on the relationship between environmental change, biodiversity loss and neglected tropical diseases (including zoonoses such as leptospirosis, rabies and over a dozen helminthiases), which have a disproportionate burden on the world’s poorest populations^238,239^. Building relationships with national public health authorities and local communities to collaboratively identify local priorities for disease control and scientific research^240^ would help to decolonize the research process and spark scientific questions. Studies on the drivers of disease emergence also substantially outnumber studies that show that these trends can actually be reversed by proposed ecological solutions: more research is needed that measures the effects of interventions such as ecosystem restoration on human and wildlife disease outcomes^209,210^. These studies will help make the case to decision-makers that ecosystem-based strategies are scientifically sound and have a high return on investment.

## Supplementary Material

**Supplementary information** The online version contains supplementary material available at https://doi.org/10.1038/s44358-024-00005-w.

Supplementary Material

## Data Availability

No original data were generated during the course of this study, but all data required to reproduce the figures here can be found at: https://github.com/viralemergence/pnpc.

## Box 1 Pandemics past and future

The distinction between a major epidemic and a pandemic is subjective, but epidemiologists generally define a pandemic as an epidemic with global spread and effects, both direct (exceptional morbidity and, potentially, mortality) and indirect (through effects on human behaviour, culture, economics, politics or well-being). Pandemics typically represent a failure of outbreak containment: a pandemic pathogen either fades after the acute period or transitions to global endemicity, making elimination impossible except as a long-term, global project. According to this definition, ten pandemics occurred during the twentieth and twenty-first centuries (Box 1 figure), most of which were responsible for at least one million deaths (Supplementary Note 3 and Supplementary Table 1).

Pandemic risks have changed throughout human history. Two bacterial pathogens — *Yersinia pestis* (plague, a vector-borne zoonosis) and *Vibrio cholerae* (cholera, a water-borne pathogen) — are collectively responsible for at least ten pandemics over the past two millennia, including the deadliest of all time (the ‘Black Death’ of the fourteenth century). However, improvements in infrastructure, sanitation, hygiene and pest control have reduced or nearly eliminated the pandemic threat from these pathogens. Today, pandemic pathogens are usually viruses, and these spread through respiratory (influenza and COVID-19) or sexual (human immunodeficiency virus) routes of transmission. Whether the next pandemic is a familiar threat (such as H5N1 influenza) or entirely new to science^242^, it is likely to entail zoonotic spillover followed by global spread through human-to-human transmission. Some groups of viruses, such as coronaviruses and paramyxoviruses, are more likely to fit this profile, owing to a high capacity for cross-species transmission and an established capacity for respiratory transmission^126^. Viruses that have originated in non-human primates (for example, human/simian immunodeficiency virus) also have a shorter evolutionary distance to close when adapting to the human immune system^44,112^.

For decades, scientists have searched for a way to predict and prevent the next pandemic^173,243,244^. Prevention efforts are most likely to succeed if they target well established pairs of pandemic threats and high-risk settings for spillover (for instance, influenza and farms or live-bird markets; coronaviruses and wildlife markets; or primate retroviruses and subsistence hunting)^100,101,156,245^. Better evidence about the drivers of disease emergence can also help to steer preparedness efforts, including the development and stockpiling of broadly effective vaccines and antivirals for the pathogens that are most likely to reappear in the coming decades^246,247^.

## Box 2 Data sources and data gaps

Ecological studies of infectious disease usually use either large-scale comparative data on host–pathogen associations or organismal-level data on infection measures, both of which suffer from data gaps and bias.

Species-level association datasets are typically assembled from published literature for the purposes of a specific study. This approach generates datasets with a high degree of overlap but several layers of taxonomic conflict and redundancy^248^.

Taxonomically harmonized, continuously updated sources such as the VIRION database^34^ have made it easier for researchers to ask questions using pre-existing, standardized data, but even these data have limitations. Most pathogens have yet to be described, and most animal and plant host species have no known pathogens recorded in these types of database (which must reflect gaps in scientific knowledge, given that nearly all forms of life have some sort of pathogen community). Outside Europe, North America, east Asia and the northern oceans, the majority of mammal species have no recorded viruses (Box 2 figure). Because of these biases, the majority of observed taxonomic or geographic variation in pathogen richness is the result of sampling effort. This limits the level of support these data can provide for hypotheses about eco-evolutionary mechanisms that explain how pathogen biodiversity has accumulated, or about the distribution of zoonotic risk across wildlife taxonomy and geographic regions^31,36,38^.

Organism-level infection datasets can be used to ask questions about the prevalence, intensity and impact of infections — all of which are more relevant to the effect of anthropogenic drivers over ecological timescales. A handful of open datasets are widely used, such as the Global Mammal Parasite Database^249^, the MalAvi database^250^ and the PREDICT project data release. These standardized datasets are generally limited by their taxonomic scope: for example, the Global Mammal Parasite Database is focused on primates, ungulates and carnivores, with no data on the two orders of mammals (rodents and bats) that contain the most species^251^. Researchers can also generate custom datasets by extracting data from the literature; meta-analyses that control for the effects of sampling effort and detection methods across studies can use these datasets to make more confident inferences about the effects of environmental drivers^233,252,253^.

At both scales, data gaps result from three overlapping problems. The first problem is genuine undersampling of some parts of the world, as well as of some wildlife taxa and traits (for example, non-synanthropic species), which can only be remedied with further sampling. Second, insufficient reporting of fine-scale spatial and temporal metadata^236^ renders many small-scale studies on pathogen detection less reusable. Third, studies with negative results — a key source of unbiased estimates of pathogen prevalence — are often either unpublished^254^, or published in less prestigious journals that can end up de-indexed by search engines (owing to predatory practices by their publishers), causing the data to become lost or less accessible for re-use. Best practices from biodiversity science could help to solve the data deficiency and quality issues in global biosurveillance: a cultural shift towards sharing raw, metadata-rich datasets at the point of publication or sooner — and new repositories such as the Pathogen Harmonized Observatory (PHAROS) that encourage the sharing of negative and unpublished data^178^ — could usher in an era in which millions or billions of data points are openly available.

## Box 3 Access and benefit-sharing

Biodiversity is a global good, but has been subject to centuries of colonial exploitation. The Convention on Biological Diversity (CBD) reaffirmed that states have a sovereign right to control the use of genetic resources originating within their borders, and that stakeholders in those resources deserve to equitably share in the benefits derived from their use. Until recently, access and benefit-sharing have been governed bilaterally under CBD and the Nagoya Protocol to the CBD (2010): each state can develop its own national implementing legislation, and sharing happens based on mutually agreed terms^255^. This framework addresses an important injustice, but researchers in the Global North have often expressed frustration about the challenges of navigating these policies, particularly when they differ between countries^256^. Disagreements also exist about how widely to define genetic resources — notably, whether this definition should include pathogens^257^ and pathogen genetic-sequence data^258^. Because of these issues, the governance of access and benefit-sharing continues to evolve: most recently, at the Conference of the Parties meeting (COP16) in 2024, CBD parties established the Cali Fund, a multilateral mechanism to facilitate the sharing of monetary benefits derived from commercial use of biodiversity.

Access and benefit-sharing are unsolved problems in global health. Scientists can only develop effective vaccines, drugs or diagnostic tests if they have access to up-to-date pathogen genetic-sequence data or physical samples from around the world. Several platforms exist to facilitate timely sharing of samples (for example, the WHO Global Influenza Surveillance and Response System) and sequence data (such as the Global Initiative on Sharing All Influenza Data database). However, Global North scientists often under-prioritize collaboration with Global South researchers who share data, and have even used urgency as a pretence for biopiracy: in a famous example, during the 2013 MERS–CoV epidemic, Dutch researchers filed for a patent on the sequence of the virus itself^259^. Moreover, once an outbreak poses a risk to high-income countries, their governments might refuse to share or sell their own supply of medical countermeasures derived from global samples; Global North companies’ monopolies on specific intellectual property (for example, messenger RNA vaccine platforms) can also prevent Global South countries from manufacturing their own goods.

These injustices were brought to the forefront during the COVID-19 pandemic^260,261^, but have been a persistent problem for longer; for example, in 2007, the Indonesian government paused sharing of influenza A/H5N1 samples, citing the inequitable sharing of benefits^262^.

The existing bilateral system under the Nagoya Protocol has achieved minimal benefits for global health. In 2011, the WHO Pandemic Influenza Preparedness Framework established a multilateral system in which vaccine manufacturers who access samples through the Global Influenza Surveillance and Response System have promised to share up to 10% of doses with the WHO during the next influenza pandemic (although this plan remains untested in practice). However, the Pandemic Influenza Preparedness Framework applies only to physical samples of influenza. In 2024, negotiations are underway among WHO member states aiming to establish a proposed pathogen access and benefit-sharing system, with broader obligations to share vaccines, drugs and diagnostics derived from genetic sequences and samples of pathogens with pandemic potential. This science-for-science exchange would be an unprecedented step towards ensuring that everyone benefits from science during public health emergencies^263^. Technical solutions and lessons learned from biodiversity informatics are helping scientists and governments to agree on a way forward that preserves essential tenets of open, rapid access to pathogen-sequence data^264^.

## Key points

- Human activities have created a planetary polycrisis that includes pandemics, climate change and the sixth mass extinction.
- Climate change, land change, agriculture and wildlife use — the major threats to biodiversity — are also driving a global rise in infectious diseases.
- Biodiversity loss is generally harmful to human health.
- Interventions that target spillover interfaces for high-risk pathogens, such as avian influenza or coronaviruses, could prevent some future pandemics.
- Even with these interventions, investments in health systems and pandemic preparedness will be an important part of living in the Anthropocene.
- The world needs better real-time biosurveillance infrastructure to track pathogens across species and ecosystems.

## Glossary

### Abbreviations

Arbovirus: A shorthand for arthropod-borne (such as mosquito-borne or tick-borne) virus.
Emerging infectious disease: An infectious disease that has recently undergone an expansion of host range, geographic range, impact or even just attention from scientific research or public health; this term is subjective, and is frequently used interchangeably with the terms zoonotic, vector-borne and environmentally transmitted diseases.
Epidemic: An infectious disease outbreak in humans, with a significant duration, size or impact.
Epizootic: An infectious disease outbreak in non-human animals, with a significant duration, size or impact.
Host competence: The ability of a host to amplify and transmit infection to another susceptible host or vector.
Macroparasite: Parasites that can be observed with the naked eye, such as ticks, fleas and some worms; the term is sometimes used interchangeably with the term parasites.
Microparasite: Microscopic parasites such as bacteria, viruses and some worms (for example, schistosomes); the term is sometimes used interchangeably with the term pathogens.
One Health: A principle that emphasizes the connections between human health, animal health and the environment, as well as the importance of solutions that benefit all three.
Pandemic: A high-impact global outbreak of a human infectious disease. This term is subjective; some sources use the term to capture any global outbreak, and others limit their definition based on the degree of circulation or mortality (see Supplementary Note 3 for all criteria we use).
Panzootic: A high-impact global outbreak of an infectious disease of animals.
Parasite: An organism that exists in an adversarial symbiotic relationship with a host.
Parasitoid: An organism that must kill a host to complete its life cycle (for example, braconid wasps).
Pathogen: An infectious microorganism that causes disease in a host.
Reservoir: Competent hosts that sustain pathogen transmission at the population or community level.
Spillback: Human-to-animal pathogen transmission (also called reverse zoonosis).
Spillover: Animal-to-human pathogen transmission.
Synanthropic: Living alongside humans (for example, in cities or human-built structures).
Vector-borne disease: A disease caused by a pathogen that is transmitted by an arthropod vector, such as a mosquito, tick or flea.
Zoonotic disease: A human infectious disease caused by a pathogen of animal origin.

## Code availability

All code developed in this study can be found at: https://github.com/viralemergence/pnpc. The minimal statistical analysis and all other data and plotting related analysis were conducted in R version 4.3.2 (2023-10-31) (R Core Team 2023) using the rstanarm package (R Core Team 2023; Goodrich et al. 2024). All other packages we used are referenced in the repository’s README file.
