# Supplementary material for: Pathogens and planetary change
Source: Nat Rev Biodivers. Author manuscript; Available in PMC 2026 Mar 4. (PMC7618822; doi:10.1038/s44358-024-00005-w)
Supplement: Supplementary Material [file EMS212026-supplement-Supplementary_Material.pdf]

nature reviews biodiversity

https://doi.org/10.1038/s44358-024-00005-w

Supplementary information

Pathogens and planetary change

In the format provided by the authors and unedited

Supplementary Information

Supplementary Note 1. Wildlife origins of emerging infectious diseases: statistics, citations, and common errors.

Summary

We encourage scientists and scientist communicators to:

1. **Stop reporting** specifically that 60% of infectious diseases, and 75% of emerging infectious diseases, are zoonotic (originate in animals), because this pair of statistics is two decades out of date, and frequently attached to the wrong language and citation.
2. Cite Jones *et al. [1]* and consider using one of these statistics, which exclude antimicrobial resistance events:
    - **Over 70% of emerging infectious diseases originate in animals**
    - **More than half of emerging infectious diseases originate in wildlife**
    - **Almost 90% of emerging viruses originate in animals**
    - **Two-thirds of emerging viruses originate in wildlife**
3. If you are interested in citing the statistics *directly* reported by Jones *et al.* [1], use these statistics below, matched to the correct language, but note that they include a significant number of antimicrobial resistance events, treated as comparable to other diseases:
    - ~60% of emerging infectious diseases originate in animals (**not 75%**)
    - ~72% (or three-quarters) of new zoonotic diseases originate in wildlife
    - ~43% of emerging infectious diseases originate in wildlife
4. In the long term, it may be worth moving away from these framings, which blur the distinction between pathogen “origins” and ongoing animal-to-human spillover. **Almost all** human infectious diseases originate in animals, but only a subset of human infectious diseases are the result of ongoing animal-to-human transmission.

Detailed explanation

Perhaps the most widely-cited study on emerging infectious diseases, Jones *et al.* (“Global trends in emerging infectious diseases,” 2008) [1], examined the first known outbreak of 335 emerging infectious diseases. Each disease is only represented once, based on its first known occurrence; this means each record represents both an outbreak and a disease, but it is not

necessarily representative of all outbreaks of a given disease. Importantly, major drug resistant lineages are counted as distinct entries from other members of the same pathogen species.

Jones *et al.* report in the main text that:

- 60.3% of all emergence events were instances of zoonotic spillover (any **animal-to-human** disease transmission).
- 71.8% of these zoonotic emergence events (i.e., 71.8% of 60.3% = 43.3% of all events) were instances of **wildlife-to-human** transmission.

In the Supplementary Information (section 1.3), they also compare their estimates to two previous studies:

- Woolhouse & Gowtage-Sequeria ("Host range and emerging and reemerging pathogens," 2005) found that out of 1,407 species of human pathogens:
    - 58% of all human infectious diseases are zoonotic
    - 73% of emerging or re-emerging infectious diseases are zoonotic
- Taylor *et al.* ("Risk factors for human disease emergence," 2001) found that out of 1,415 species of human pathogens:
    - 61% of all human infectious diseases are zoonotic
    - 75% of emerging infectious diseases are zoonotic
- Both of these studies find that roughly 60% of infectious diseases, and 75% of emerging infectious diseases, are zoonotic; in contrast, Jones et al. do not examine non-emerging infections, and find that only 60% of emerging infectious diseases are zoonotic.

Problem 1: Citations are often confused or mixed

Numbers from the three studies are frequently mixed and incorrectly attributed to Jones *et al.,* sometimes with the wrong descriptors attached. "60%" and "75%" frequently appear back to back; for example, a UNEP report on "Preventing the Next Pandemic" states that:

> About 60 per cent of human infections are estimated to have an animal origin [**citing Woolhouse**], and of all new and emerging human infectious diseases, some 75 per cent "jump species" from (non-human) animals to people [**citing Taylor**].

In this case, the citations are correct and matched to the correct statistics and explanations. In contrast, the World Health Organization's fact sheet on One Health reports that:

> Some 60% of emerging infectious diseases that are reported globally come from animals, both wild and domestic [**This statistic - 60% of EIDs are zoonotic - is from Jones 2008**]. Over 30 new human pathogens have been detected in the last 3 decades, 75% of which have originated in animals [**This statistic - 75% of EIDs are zoonotic - is from Taylor 2001, and as written, contradicts the first statement**].

The statistic that 75% of emerging infectious diseases originate in animals is over two decades out of date, and is superseded by Jones *et al.*, who found a much lower number.

Problem 2: Jones *et al.* included antimicrobial resistance events.

Out of 335 events, 70 (20.9%) are reported by Jones as representing the emergence of drug resistance. This statistic is, itself, apparently incorrect due to errors in the data; a dozen drug-resistant pathogens are incorrectly reported as a "0" in the DrugRes field:

1. Human Herpesvirus 3 acyclovir-res
2. *Mycobacterium bovis* multiple drug-res
3. *Mycobacterium tuberculosis* isoniazid-res
4. *Neisseria gonorrhoeae* fluoroquinolones-res
5. *Neisseria gonorrhoeae* penicillin-res
6. *Plasmodium falciparum* chloroquine-res
7. *Plasmodium falciparum* mefloquine-res
8. *Plasmodium falciparum* multiple drug-res
9. *Plasmodium falciparum* proguanil-res
10. *Plasmodium falciparum* quinine-res
11. *Plasmodium falciparum* sulfadoxine-pyrimethamine-res
12. *Plasmodium vivax* chloroquine-res

The 82 drug resistance emergence events represent the origin of new genotypes, rather than new pathogens. After excluding them from the sample:

- 184 of 253 emerging infectious diseases are zoonotic (72.7%, compared to 60.3%; i.e., the major difference from Taylor or Woolhouse is the inclusion of these events).
- Of these, 141 (76.6%) of zoonotic diseases have a wildlife origin; in total, 55.7% of all emerging infectious diseases originate in wildlife.
- 58 of 76 emerging viruses (76.3%) are zoonotic. Of them, 51 originate in wildlife (87.9%). 67% of all emerging viruses originate in wildlife.

Problem 3: Almost all human infectious diseases have an evolutionary origin in animals.

The idea that only a fraction of human infectious diseases "originate" in animals is associated with a set of tacit distinctions about ecological versus evolutionary timescales, which are rarely communicated when these statistics are cited.

When studies make a list of human infectious diseases, and identify the subset that are zoonotic in origin, they are usually referring to origins on the timescale of specific and recent transmission events (e.g., Hendra virus is only able to be a "human virus" because it is periodically transmitted

from bats to humans). Many human pathogens do not currently spread between animals and humans, but with very rare exceptions, almost all human pathogens are a recent branch of a much deeper evolutionary tree. As animal viruses become better characterized, it is increasingly obvious that nearly all human viruses originated in animal-infective clades [2]. For example, in 2020, the first close relatives of rubella virus were reported: ruhugu virus (discovered in African bats) and rustrela virus (found in captive marsupials and free-living mice in a European zoo) [3]. Exceptions to this pattern are very rare: for example, the Victoria and Yamagata lineages of influenza B virus likely diverged from a common ancestor in humans in the 1970s [4].

The distinction between the ecological and evolutionary timescales that make a "zoonosis" is ultimately subjective. For example, HIV is typically treated as a zoonotic virus (although, perhaps in error, Jones *et al.* categorize HIV-1 zoonotic, but not HIV-2), while measles is not; however, HIV diverged from simian immunodeficiency viruses after several spillover events during the early- to- mid-20th century [5,6], while measles virus diverged from rinderpest virus (a cattle-exclusive pathogen) following a spillover event sometime around the 6th Century [7]. Both are the result of animal-to-human disease transmission events during recorded history.

These distinctions may seem trivial or pedantic, but they highlight an ongoing way that these often-cited statistics are used to both over- and under-state the importance of zoonotic risk. On one hand, a much higher proportion of human infectious diseases "originate" in animals than 60%. On the other, only a subset of the diseases that are generally considered "zoonotic" are actively transmitted between wildlife and humans, while others (like HIV) are now entirely human pathogens. Over time, the field could move away from using these statistics, and instead use two distinct framings: almost all new human infectious diseases originate in animals, but a significant proportion of human diseases are the result of ongoing animal-to-human transmission.

Supplementary Note 2. Data and methods underlying Figure 1.

Panel A: Data are sourced from a previous study on the spillover of high-impact zoonotic viruses [8]. The fitted line is the expected predicted values from a Bayesian negative-binomial GLM, with associated credible intervals.

Panel B: This plot is a recreation of Figure 1a from[9], which shows estimated cumulative extinction rates of various vertebrate taxa through time, relative to a background rate. We extracted these data from the supplementary information. Note that here we plot the x-axis as the date range midpoints, but the data originally are referenced as the full ranges: 1500-1600, 1600-1700, 1700-1800, 1800-1900, 1900-2010.

Panel C: Instrumental temperature data (doi: 10.25921/xq6s-hf81) and atmospheric CO_2 level data (doi: 10.15138/9N0H-ZH07) were provided by NOAA. Climate reconstruction data are taken from [10]. Temperature anomalies are plotted on the y-axis, with data from ensemble re-constructions up to 1850 (gray line with 95% ensemble confidence intervals), and instrumental data from 1850 to 2017. Anomalies are given relative to a baseline of 1600-1699 (rebaselined from the source, which was baselined in the mid-1900s). The color of the instrumental data post-1850 represents the parts-per-million of atmospheric CO_2 .

Panel D: Mammal zoonotic host richness was mapped by combining IUCN Red List range maps and the expanded CLOVER dataset of vertebrate-pathogen interactions, excluding domestic species [11]. A zoonotic host is defined as any mammal species that is known to harbor at least one pathogen also known to infect humans. Emerging infectious disease event data were taken from EIDR (https://eidr.ecohealthalliance.org/event-map), and were updated through 2013. Points are coded by pathogen type.

More information and supplementary details about how these plots were created and which data sources were used can be found on GitHub:
https://github.com/viralemergence/pnpc/blob/main/data/recreation/README.md

Supplementary Note 3. Methods underlying Box 1.

What we excluded from our definition of pandemics.

We identified ten pandemics that have happened, at least partly, since the year 1900. Several other major outbreaks are often included in similar lists, but are excluded from a strict definition of "pandemic" based on one of two reasons.

The first is **limited geographic scope**. For example, the Zika virus epidemic in the Americas (2015-2017; ~750,000 reported cases in the Americas [12]) has had an undeniable impact, but – although autochthonous and imported cases were reported in over 80 countries [12] – the acute epidemic, and the impact of Zika congenital syndrome, was mostly limited to the Americas. Other regional epidemics that fall short of our definition include the chikungunya virus epidemic in the Americas (2013-16; 3.7 million cases reported by 2023 [13]) and outbreaks of Ebola virus in west Africa (2013-2016; ~29,000 cases) and central Africa (2018-2020; ~3,500 cases).

The second reason is **limited spread in the general population**, sometimes (but not always) due to successful containment. For example, the 2002 SARS-CoV outbreak could potentially have become a Covid-19-like pandemic, but was contained through contact tracing, isolation, and quarantine (~8,000 cases total) [14]. Acute global outbreaks of both MERS-CoV (~2,600 cases since 2012) and clade II mpox (over 100,000 cases since 2022) were mostly contained through the same approach (and, in the latter case, vaccination), although spillover and spread of both have continued. This criterion similarly disqualifies the 1929 psittacosis "pandemic" (750-800 cases) [15] and the 2022 global outbreak of viral hepatitis in children (>1,000 probable cases) [16].

The ten pandemics.

Based on these criteria for inclusion and exclusion, we identified a total of ten pandemics that have taken place at least partly since the year 1900. For each, we used our expert opinion and a non-systematic review of the literature to assign origin and drivers, and provided a best possible estimate of the number of resulting deaths. Sources are given in Supplementary Table 1, on the following page.

Supplementary Table 1. Pandemic origins.

Disease	Timing	Deaths	Origin	Drivers			
				Agriculture	Habitat loss	Wildlife use	Climate change
Plague	1855-1960	12-15 million [17]	Rats, fleas [18]				
Cholera (O1 classic biotype)	1899-1923	> 1.5 million [19,20]	Water				
Influenza (A/H1N1)	1918-1920	> 50 million [21]	Birds [22,23]	[22,23]			
Influenza (A/H2N2)	1957-1958	1.1 million [24]	Birds [22,23]	[22,23]			
Cholera (O1 El Tor biotype)	1961-	~95,000 / year at most recent estimate [25]	Water				[26]
Influenza (A/H3N2)	1968-1969	1-4 million [27]	Birds [22,23]	[22,23]			
Influenza (A/H1N1)	1977-1979	0.7 million [28]	Likely not natural, possibly related to vaccine trials [29,30]				
HIV/AIDS	1981-	42 million [31]	Non-human primates [5]			[32]	
Influenza (A/H1N1)	2009-2010	123,000-203,000 [33]	Swine [34]	[34]			
Covid-19	2020-	18 million [35]	Proximal: potentially raccoon dogs [36]; ultimate: probably bats [37,38]			[36,39,40]	

Supplementary References

1. Jones KE, Patel NG, Levy MA, Storeygard A, Balk D, Gittleman JL, et al. Global trends in emerging infectious diseases. Nature. 2008;451: 990–993.

2. Lu L, Zhang F, Brierley L, Robertson G, Chase-Topping M, Lycett S, et al. Temporal Dynamics, Discovery, and Emergence of Human-Transmissible RNA Viruses. Mol Biol Evol. 2024;41. doi:10.1093/molbev/msad272

3. Bennett AJ, Paskey AC, Ebinger A, Pfaff F, Priemer G, Höper D, et al. Relatives of rubella virus in diverse mammals. Nature. 2020;586: 424–428.

4. Virk RK, Jayakumar J, Mendenhall IH, Moorthy M, Lam P, Linster M, et al. Divergent evolutionary trajectories of influenza B viruses underlie their contemporaneous epidemic activity. Proc Natl Acad Sci U S A. 2020;117: 619–628.

5. Wertheim JO, Worobey M. Dating the age of the SIV lineages that gave rise to HIV-1 and HIV-2. PLoS Comput Biol. 2009;5: e1000377.

6. Sharp PM, Hahn BH. The evolution of HIV-1 and the origin of AIDS. Philos Trans R Soc Lond B Biol Sci. 2010;365: 2487–2494.

7. Düx A, Lequime S, Patrono LV, Vrancken B, Boral S, Gogarten JF, et al. Measles virus and rinderpest virus divergence dated to the sixth century BCE. Science. 2020;368: 1367–1370.

8. Meadows AJ, Stephenson N, Madhav NK, Oppenheim B. Historical trends demonstrate a pattern of increasingly frequent and severe spillover events of high-consequence zoonotic viruses. BMJ Glob Health. 2023;8. doi:10.1136/bmjgh-2023-012026

9. Ceballos G, Ehrlich PR, Barnosky AD, García A, Pringle RM, Palmer TM. Accelerated modern human-induced species losses: Entering the sixth mass extinction. Sci Adv. 2015;1: e1400253.

10. PAGES 2k Consortium. Consistent multidecadal variability in global temperature reconstructions and simulations over the Common Era. Nature Geoscience. 2019;12: 643–649.

11. Gibb R, Albery GF, Becker DJ, Brierley L, Connor R, Dallas TA, et al. Data Proliferation, Reconciliation, and Synthesis in Viral Ecology. Bioscience. 2021;71: 1148–1156.

12. Hills SL, Fischer M, Petersen LR. Epidemiology of Zika Virus Infection. J Infect Dis. 2017;216: S868–S874.

13. de Souza WM, Ribeiro GS, de Lima STS, de Jesus R, Moreira FRR, Whittaker C, et al. Chikungunya: a decade of burden in the Americas. Lancet Reg Health Am. 2024;30:

100673.

14. Bell DM, World Health Organization Working Group on International and Community Transmission of SARS. Public health interventions and SARS spread, 2003. Emerg Infect Dis. 2004;10: 1900–1906.

15. Ramsay EC. The Psittacosis Outbreak of 1929–1930. J Avian Med Surg. 2003;17: 235–237.

16. Ho A, Orton R, Tayler R, Asamaphan P, Herder V, Davis C, et al. Adeno-associated virus 2 infection in children with non-A–E hepatitis. Nature. 2023;617: 555–563.

17. Echenberg M. Pestis redux: the initial years of the third bubonic plague pandemic, 1894-1901. J World Hist. 2002;13: 429–449.

18. Bramanti B, Dean KR, Walløe L, Chr Stenseth N. The third plague pandemic in Europe. Proc Biol Sci. 2019;286: 20182429.

19. Rogers L. The Conditions Influencing the Incidence and Spread of Cholera in India. Proc R Soc Med. 1926;19: 59–93.

20. Ramamurthy T, ICMR-National Institute of Cholera and Enteric Diseases P-33 C.I.T. Road, Scheme-XM, Beliaghata Kolkata 700010, West Bengal, India, Ghosh A. A re-look at cholera pandemics from early times to now in the current era of epidemiology. J Disaster Res. 2021;16: 110–117.

21. Johnson NPAS, Mueller J. Updating the accounts: global mortality of the 1918-1920 "Spanish" influenza pandemic. Bull Hist Med. 2002;76: 105–115.

22. Nelson MI, Worobey M. Origins of the 1918 pandemic: Revisiting the swine "mixing vessel" hypothesis. Am J Epidemiol. 2018;187: 2498–2502.

23. Wille M, Holmes EC. The Ecology and Evolution of Influenza Viruses. Cold Spring Harb Perspect Med. 2020;10. doi:10.1101/cshperspect.a038489

24. Viboud C, Simonsen L, Fuentes R, Flores J, Miller MA, Chowell G. Global Mortality Impact of the 1957-1959 Influenza Pandemic. J Infect Dis. 2016;213: 738–745.

25. Ali M, Nelson AR, Lopez AL, Sack DA. Updated global burden of cholera in endemic countries. PLoS Negl Trop Dis. 2015;9: e0003832.

26. Colwell RR. Global climate and infectious disease: the cholera paradigm. Science. 1996;274: 2025–2031.

27. Honigsbaum M. Revisiting the 1957 and 1968 influenza pandemics. Lancet. 2020;395: 1824–1826.

28. Burke DS, Schleunes A. A self-fulfilling prophecy pandemic: The 1977 "Russian flu."

Perspect Biol Med. 2024;67: 386–405.

29. Rozo M, Gronvall GK. The reemergent 1977 H1N1 strain and the gain-of-function debate. MBio. 2015;6. doi:10.1128/mBio.01013-15

30. Palese P. Influenza: old and new threats. Nat Med. 2004;10: S82–7.

31. World Health Organization. HIV. In: WHO Global Health Observatory [Internet]. 2024 [cited 29 Sep 2024]. Available: https://www.who.int/data/gho/data/themes/hiv-aids

32. Rupp S, Ambata P, Narat V, Giles-Vernick T. Beyond the cut Hunter: A historical epidemiology of HIV beginnings in central Africa. Ecohealth. 2016;13: 661–671.

33. Simonsen L, Spreeuwenberg P, Lustig R, Taylor RJ, Fleming DM, Kroneman M, et al. Global mortality estimates for the 2009 Influenza Pandemic from the GLaMOR project: a modeling study. PLoS Med. 2013;10: e1001558.

34. Smith GJD, Vijaykrishna D, Bahl J, Lycett SJ, Worobey M, Pybus OG, et al. Origins and evolutionary genomics of the 2009 swine-origin H1N1 influenza A epidemic. Nature. 2009;459: 1122–1125.

35. Wang H, Paulson KR, Pease SA, Watson S, Comfort H, Zheng P, et al. Estimating excess mortality due to the COVID-19 pandemic: a systematic analysis of COVID-19-related mortality, 2020–21. Lancet. 2022;399: 1513–1536.

36. Crits-Christoph A, Levy JI, Pekar JE, Goldstein SA, Singh R, Hensel Z, et al. Genetic tracing of market wildlife and viruses at the epicenter of the COVID-19 pandemic. Cell. 2024;187: 5468–5482.e11.

37. Pekar JE, Lytras S, Ghafari M, Magee AF, Parker E, Havens JL, et al. The recency and geographical origins of the bat viruses ancestral to SARS-CoV and SARS-CoV-2. bioRxivorg. 2023. doi:10.1101/2023.07.12.548617

38. Lytras S, Hughes J, Martin D, Swanepoel P, de Klerk A, Lourens R, et al. Exploring the natural origins of SARS-CoV-2 in the light of recombination. Genome Biol Evol. 2022;14. doi:10.1093/gbe/evac018

39. Worobey M, Levy JI, Malpica Serrano L, Crits-Christoph A, Pekar JE, Goldstein SA, et al. The Huanan Seafood Wholesale Market in Wuhan was the early epicenter of the COVID-19 pandemic. Science. 2022;377: 951–959.

40. Pekar JE, Magee A, Parker E, Moshiri N, Izhikevich K, Havens JL, et al. The molecular epidemiology of multiple zoonotic origins of SARS-CoV-2. Science. 2022;377: 960–966.
